# Gut microbiota, innate immune pathways, and inflammatory control mechanisms in patients with major depressive disorder

**DOI:** 10.1038/s41398-021-01755-3

**Published:** 2021-12-21

**Authors:** Javier R. Caso, Karina S. MacDowell, Ana González-Pinto, Saínza García, Javier de Diego-Adeliño, Mar Carceller-Sindreu, Fernando Sarramea, Javier Caballero-Villarraso, Patricia Gracia-García, Concepción De la Cámara, Luis Agüera, María L. Gómez-Lus, Claudio Alba, Juan M. Rodríguez, Juan C. Leza

**Affiliations:** 1 Department of Pharmacology & Toxicology, Faculty of Medicine, Universidad Complutense de Madrid (UCM), Madrid, Spain; 2 Centro de Investigación Biomédica en Red de Salud Mental (CIBERSAM), Instituto de Investigación Hospital 12 de Octubre (Imas12), IUIN-UCM, Madrid, Spain; 3 University Hospital of Alava (HUA), University of the Basque Country (UPV/EHU), CIBERSAM, Vitoria-Gasteiz, Spain; 4 Department of Psychiatry, Hospital de la Santa Creu i Sant Pau, Institut d’Investigació Biomèdica Sant Pau (IIB-Sant Pau), Universitat Autònoma de Barcelona (UAB), CIBERSAM, Barcelona, Spain; 5 Instituto Maimónides de Investigación Biomédica de Córdoba (IMIBIC), Unidad de Gestión Clínica de Salud Mental, Hospital Universitario Reina Sofía, CIBERSAM, Córdoba, Spain; 6 Servicio de Psiquiatría, Hospital Universitario Miguel Servet, Departamento de Medicina y Psiquiatría, Universidad de Zaragoza, CIBERSAM, Zaragoza, Spain; 7 Servicio de Psiquiatría, Hospital Clínico Universitario, Departamento de Medicina y Psiquiatría, Universidad de Zaragoza, CIBERSAM, Zaragoza, Spain; 8 Psychiatry Department, Hospital Universitario 12 de Octubre, UCM, CIBERSAM, Madrid, Spain; 9 Department of Medicine—Microbiology Area, Faculty of Medicine, UCM, Madrid, Spain; 10 Department of Nutrition and Food Science, Faculty of Veterinary, UCM, Madrid, Spain

**Keywords:** Depression, Biomarkers

## Abstract

Although alterations in the gut microbiota have been linked to the pathophysiology of major depressive disorder (MDD), including through effects on the immune response, our understanding is deficient about the straight connection patterns among microbiota and MDD in patients. Male and female MDD patients were recruited: 46 patients with a current active MDD (a-MDD) and 22 in remission or with only mild symptoms (r-MDD). Forty-five healthy controls (HC) were also recruited. Psychopathological states were assessed, and fecal and blood samples were collected. Results indicated that the inducible nitric oxide synthase expression was higher in MDD patients compared with HC and the oxidative stress levels were greater in the a-MDD group. Furthermore, the lipopolysaccharide (an indirect marker of bacterial translocation) was higher in a-MDD patients compared with the other groups. Fecal samples did not cluster according to the presence or the absence of MDD. There were bacterial genera whose relative abundance was altered in MDD: *Bilophila* (2-fold) and *Alistipes* (1.5-fold) were higher, while *Anaerostipes* (1.5-fold) and *Dialister* (15-fold) were lower in MDD patients compared with HC. Patients with a-MDD presented higher relative abundance of *Alistipes* and *Anaerostipes* (1.5-fold) and a complete depletion of *Dialister* compared with HC. Patients with r-MDD presented higher abundance of *Bilophila* (2.5-fold) compared with HC. Thus, the abundance of bacterial genera and some immune pathways, both with potential implications in the pathophysiology of depression, appear to be altered in MDD, with the most noticeable changes occurring in patients with the worse clinical condition, the a-MDD group.

## Introduction

Inflammatory processes can be implicated in the development of depressive-like symptoms or major depressive disorder (MDD) [1–3]. It appears that MDD and inflammation are fueling each other: inflammation promoting depression and MDD facilitating inflammatory reactions [4]. MDD patients usually exhibit alteration of biomarkers of immune dysfunction and data point toward the manifestation of all components of an archetypical inflammatory response in MDD [4]. Besides, inflammation is linked with the clinical severity of mood disorders and remission of MDD is connected with the normalization of inflammatory markers [5, 6].

Damage-associated molecular patterns (DAMPs) are endogenous molecules whose production is increased after stressor exposure and tissue damage [7]. In the nonexistence of tissue damage, DAMPs induce the so-called systemic sterile inflammation [8]. Despite some evidence of increased levels of peripheral DAMPs in neuropsychiatric diseases (e.g., heat shock proteins and high-mobility group box 1 (HMGB1)) [7, 9], more studies are required to verify the participation of DAMPs in prompting chronic low-grade inflammation in these pathologies. Consequently, the pattern recognition receptors (PRRs) are getting attention. In the central nervous system (CNS) PRRs are primarily expressed by microglia, macrophages and astrocytes and they are either membrane-bound (Toll-like receptors (TLRs)) or found within the cytoplasm (nucleotide-binding oligomerization domain-like receptors) sensing intracellular signals [10, 11].

Ligands of PRRs include those related to damage (DAMPs) and pathogens (pathogen-associated molecular patterns (PAMPs)) and those related to microbes (microbe-associated molecular patterns (MAMPs)) comprising both pathogenic and commensal/symbiotic microbes [7]. The TLRs potential role in the pathogenesis of psychiatric diseases is being intensely studied [9]. The most studied is TLR-4, which responds not only to lipopolysaccharide (LPS), a component of the outer membrane of Gram-negative bacteria, but also to DAMPs, resulting in the activation of inflammatory transcription factors [12, 13].

Preclinical studies indicate a substantial similarity in numerous inflammatory parameters that are modulated after either LPS or stressor exposure, which are detectable in the blood and peripheral tissues [7, 9]. Patterns from gut bacteria can be considered MAMPs or PAMPs, depending on the setting, and they can induce an immune activation in the CNS [14, 15]. Consequently, it is worth to investigate the relationship between the microbiota–gut–brain (MGB) axis and the immune response ongoing in MDD, especially considering the lack of understanding about the connection among microbiota, immune response and MDD [16, 17].

Microbiota impacts numerous aspects of physiology [18], and data from experimental animal models and preclinical studies reveal that depression could influence microbiota composition and suggest that gut microbiota might impact brain function and behavior through the MGB axis [19, 20]. Thus, gut microbiota alterations could be a contributory factor to the development of MDD [21]. These alterations would increase the intestinal barrier permeability allowing a bacterial translocation [22], which could be related to the inflammatory hypothesis of depression [23]. Animal studies show that experimental models of depression affect the intestinal barrier allowing the bacteria translocation and inducing a neuroinflammatory response through TLR-4 activation in the brain [24, 25]. This scenario seems to happen in MDD patients as well, suggesting that bacterial translocation would be related to the inflammatory pathophysiology of the MDD [26, 27].

Studies examining gut microbiota in MDD and healthy controls (HC) generally show decreased microbial richness and diversity [28]. A research has studied active and remitted MDD compared to controls showing differences in diversity, and in the levels of specific bacterial taxa, particularly among patients with clinically significant depressive symptoms [29]. However, the association between gut microbiota and MDD, and between microbiota and inflammatory markers/processes persists weakly comprehended. An assessment of the fecal microbiota of patients with active MDD (a-MDD) and remained in remission or with only mild symptoms (r-MDD) compared with HC and the possible actions on the immune response should be undertaken before drawing definite conclusions; these are the main aim and justification of our study, in which we have shown variations of bacteria genera and the activation of proinflammatory pathways, that can be indirectly related to bacterial translocation, with the most noticeable changes occurring in a-MDD patients.

Employing samples from MDD patients (including a-MDD and r-MDD patients) and HC, the aims of this study were: (a) identifying whether human fecal microbiota is altered during MDD compared with HC; (b) detecting microbiota signatures distinctive for MDD and their connections with the possible inflammatory scenarios present in patients compared with HC; (c) recognizing TLR-4 and proinflammatory pathways activation in blood from MDD patients compared with HC; and (d) finding whether the possible scenarios detected by the previous points are also happening among a-MDD and r-MDD patients as well as compare them with HC.

## Materials/Subjects and methods

### Participants

Outpatients from multiple hospitals in different cities, with a documented Diagnostic and Statistical Manual of Mental Disorders, 4th ed., Text Revision (DSM-IV-TR) [30] diagnosis of MDD, through structured clinical interviews performed by psychiatrists, participated in the study. An HC group with a similar age and sex distribution to the patients was recruited, and clinically evaluated in the same manner as patients, among their relatives and graduate students and/or hospital staff. Procedures were approved by the Hospitals’ Review Boards and the Complutense Ethics Committee (project number PI13/01102). Procedures comply with Spain’s legislation (Ley 14/2007) and the Helsinki Declaration. All participants signed a written informed consent after receiving a complete description of the study. Inclusion/exclusion criteria are described in Supplementary Material.

The study included 68 MDD patients and 45 HC. The MDD sample was divided into two groups for additional analyses: one composed by patients with a current active depressive episode according to DSM-IV-TR and a Hamilton Depression Rating Scale (HDRS) > 14 (a-MDD) and a separate group of participants composed by patients who had responded to treatment and remained in remission or with only mild symptoms (HDRS 8–14) (r-MDD).

### Clinical assessments

An ad hoc protocol was used for demographic and clinical characteristics. Clinical and psychopathological states were assessed by means of the Spanish versions of the HDRS [31], the Euroquol-5D visual analog scale (EQ-Vas, a measure of self-perceived health-related quality of life) [32], the perceived stress scale (PSS) [33], list of threatening experiences questionnaire (LTE-Q) [34], and the childhood trauma questionnaire-short form (CTQ-SF) [35]. Rome III criteria for functional gastrointestinal pathology [36] in the last 3 months were evaluated in all the groups by means of a questionnaire (ten yes/no items) while the psychological assessments were performed.

Data were analyzed using the D’Agostino and Pearson test to assess Gaussian distribution. An unpaired two-tailed *t*-test was performed when there were no values in the HC group and a one-way ANOVA with a Tukey post-hoc test was employed for comparisons between the HC, a-MDD, and r-MDD groups. When data did not follow a Gaussian distribution, a nonparametric ANOVA with a Kruskal–Wallis and a Dunn’s post-hoc test was performed.

### Metataxonomic analysis of the fecal samples

#### Sample collection and DNA extraction

All metataxonomic analysis were performed by investigators totally blinded to the groups allocation. Fecal samples were collected in a sterile plastic cup after the participants completed the clinical assessments and were kept in an icebox. Samples for bacterial genomic DNA extraction were delivered to the laboratory and stored at −80 °C, thus, samples were kept on ice for not longer than 1 h. Microbial DNA was extracted from fecal aliquots (200 mg) using the QIAamp^©^ DNA Stool Mini Kit (Qiagen, Hilden, Germany) according to the manufacturer’s instructions, with the additional glass-bead beating steps on a Mini-beadbeater (FastPrep; Thermo Electron Corp., Boston, MA, USA). DNA was quantified using a NanoDrop ND-1000 spectrophotometer (Thermo Electron); integrity and size were assessed by 1.0% agarose gel electrophoresis on gels containing 0.5 mg/mL ethidium bromide. DNA was stored at 20 °C before analysis and all samples were processed and sequenced in the same batch to avoid impacts in the microbial results.

#### PCR amplification and sequencing

16S rDNA gene amplicons were amplified following the Illumina protocol for 16S rDNA gene metagenomic sequencing library preparation (part# 15044223 Rev. A). The forward primer (TCGTCGGCAGCGTCAGATGTGTATAAGAGACAGCCTACGGGNGGCWGCAG) and the reverse one (TACGGTAGCAGAGACTTGGTCTGACTACHVGGGTATCTAATCC) were used as previously described [37], generating amplicons targeting the V3-V4 hypervariable region of the 16S rDNA gene. An aliquot of the microbial DNA from each sample (5 ng/μL in 10 mM Tris pH 8.5) was used to initiate the protocol. Libraries were sequenced using a 2 × 300 bp paired-end run (MiSeq Reagent kit v3, MS-102-3001) on a MiSeq Sequencer according to manufacturer’s instructions (Illumina, USA).

#### Quality assessment and taxonomy

Quality assessment was performed using prinseq-lite program [38]. R1 and R2 from Illumina sequencing were joined using fastq-join from ea-tools suite [39]. The amplified fragments were clustered in operational taxonomic units (OTUs) and representative sequences were taxonomically analyzed using RDP_classifier from the Ribosomal Database Project [40]. Raw datasets are available in the E-Prints Complutense repository, ID Code: 64666.

#### Bioinformatic analysis

The bioinformatic analysis was conducted combining R (v 3.2.3), QIIME pipelines (v 1.8.0) [41], and Calypso (v 8.84) [42]. Estimates of intrasample diversity were made at a rarefaction depth of 27,000 reads per sample. Alpha diversity was assessed with the Shannon diversity index, which considers the number and evenness of microbial species. Differences between groups were assessed using either Kruskal–Wallis tests for three groups comparison (control, a-MDD, and r-MDD) or Wilcoxon rank sum tests for pairwise and two groups (control and MDD) comparisons. Distance matrices containing a dissimilarity value for each pairwise sample comparison were performed to evaluate the beta diversity and to plot patterns of bacterial community diversity. For the quantitative (relative abundance) and qualitative (presence/absence) analyses, the Bray–Curtis dissimilarity and binary Jaccard distance indices were used, respectively. Principal coordinates analysis (PCoA) was used to visually display patterns of beta diversity through the distance matrices. The PERMANOVA analysis with 999 permutations was performed to reveal statistically significant differences (*p* < 0.05). Differences in genera were compared by using either Kruskal–Wallis tests for variables with three groups comparison (control, a-MDD, and r-MDD) or Wilcoxon rank sum tests for pairwise and control vs. MDD comparisons.

To correct for multiple comparisons, Bonferroni-adjusted significance levels were set for each analysis. The bar chart with the cladogram was performed with the Hclust hierarchical cluster analysis with complete linkage method from the Calypso online software.

### Biochemical determinations in plasma and peripheral blood mononuclear cells (PBMCs)

#### Specimen collection and preparation

Venous blood samples (10 mL) were collected in the morning, between 8 a.m. and 10 a.m., after overnight fasting. Blood tubes were centrifuged (641 × *g* for 10 min at 4 °C). Plasma samples were collected and stored at −80 °C. The rest of the sample was 1:2 diluted in culture medium (RPMI 1640, LifeTech) and a gradient with Ficoll-Paque (GE Healthcare) was used to isolate mononuclear cells by centrifugation (800 × *g* for 40 min at room temperature [RT]). The PBMC layer was aspired, suspended in RPMI and centrifuged (1116 × *g* for 10 min at RT). The supernatant was removed, and the mononuclear cell-enriched pellet was stored at −80 °C.

#### Determinations in plasma

Biochemical parameters in plasma were measured using commercially available kits and following the manufacturers’ instructions (details in Supplementary Material).

#### Determinations in PBMCs

PBMC samples were fractionated in cytosolic and nuclear extracts using a method which provides a high purity nuclear fraction [43]. Protein expression analyses were performed in cytosolic extracts, except for the two transcription factors studied (i.e., NF-κBp65 and PPAR_γ_), whose expression levels were analyzed in nuclear extracts (details in Supplementary Material). Antibodies’ identifiers provided by the Resource Identification Portal can be seen in Supplementary Table S1.

### Statistical analysis of the biochemical determinations

Data are expressed as mean ± standard error of the mean. The ROUT method was used to identify outliers. Data were analyzed using the D’Agostino and Pearson test to assess Gaussian distribution. First, the whole group of MDD patients was compared with HC: an unpaired two-tailed *t*-test was performed. Second, a one-way ANOVA with a Tukey post-hoc test was employed for comparisons between the HC, a-MDD, and r-MDD groups. When the data did not follow a Gaussian distribution, a nonparametric ANOVA with a Kruskal–Wallis test followed by a Dunn’s post-hoc test was performed. The variance was similar among the groups. A *p* value < 0.05 was considered statistically significant in all cases. ANOVAs’ statistical details can be observed in Supplementary Table S2.

## Results

### Demographic and clinical characteristics of the sample

Patients were white Caucasian and most of the participants were married and had secondary or primary education. The mean age was 43.98 years and 77.77% females, and 46 patients were a-MDD (mean age 42.1 years, 78.26% females) and 22 were r-MDD (mean age 45.85 years, 77.27% females). These groups were compared with 45 HC (mean age 44.72 years, 75.5% females). There were no differences among the groups in age nor in gender. The Rome III criteria indicated that none of the participants in the study presented functional gastrointestinal disorders. All participants presented a body mass index under 30.

The average number of months since the onset of the disease was 125.73 in a-MDD and 160.91 in r-MDD patients.

The a-MDD group showed higher HDRS, PSS, and LTE-Q scores than the r-MDD group. The r-MDD group showed higher levels in the EQ-Vas scale and, consequently, a higher health-related quality of life than patients with a-MDD (66.58 vs. 38.78, respectively) although their values were still far from the ones in the HC group (89.82). Both MDD groups had higher scores in the childhood trauma scales when compared with HC (Table 1).

**Table 1:** Sociodemographic and clinical characteristics of the subjects.

	Healthy controls (HC), *n* = 45	Acute MDD (a-MDD), *n* = 46	Remission MDD (r-MDD), *n* = 22
Sex (women), *n* (%)	34 (75.5%)	36 (78.26%)	17 (77.27%)
Age (years)	44.72	42.10	45.85
Employment status			
Full-time job	34	20	9
Part-time job	0	0	0
Student	3	1	1
Unemployed	5	8	9
Retired	1	1	2
On sick leave	2	16	1
N/A	0	0	0
Educational level			
Primary education	16	14	14
Secondary education	10	18	3
University studies	18	13	4
N/A	1	2	1
Relationship status			
Single	12	10	6
Married (or solid relationship)	24	27	13
Divorced	8	5	2
N/A	1	4	1
Onset of the disease (months)	n.a.	125.73	**160.91**^###^
Previous treatments			
On antidepressant, *n* (%)	0	38 (82.6)	20 (90.9)
On others, *n* (%)	0	17 (36.9)	2 (9.09)
HDRS	0.08	21.17***	10.54**^,###^
EQ-Vas	89.82	38.78***	66.58***^,###^
PSS	19.47	33.82***	27.35***^,###^
LTE-Q	0.81	1.26***	1.02*
Childhood trauma			
CTQ-SF total scores	28.74	40.19***	46.22***^,#^
CTQ-EA (emotional abuse)	5.27	8.9***	8.81***^,##^
CTQ-PA (physical abuse)	5.22	6.58***	6.26***
CTQ-SA (sexual abuse)	5.04	6.77***	6.4***^,##^
CTQ-EN (emotional neglect)	6.79	10.38***	10.63***^,##^
CTQ-PN (Physical neglect)	5.35	7.28***	7.53***^,##^
Functional gastrointestinal pathology (Rome III criteria)	Negative	Negative	Negative
Smoking, cig/day (*n* users)	14 (11)	9.8 (19)	6.36 (5)
Alcohol, SDU/week (*n* users)	9.7 (22)	2.4 (11)	1.75 (4)
Other (*n* users)	1	2	0

An unpaired two-tailed *t*-test was performed when comparing two groups. For more than two groups comparisons, a one-way ANOVA with a Tukey post-hoc test was employed, and in those cases in which the data did not follow a Gaussian distribution, a nonparametric ANOVA with a Kruskal–Wallis test followed by a Dunn’s post-hoc test was performed. Bold values indicates statistical significant *p* values. *p < 0.05, **p < 0.01, ***p < 0.001 vs. HC; ^#^*p* < 0.05, ^##^*p* < 0.01, ^###^*p* < 0.001 vs. a-MDD.

*HDRS* Hamilton Depression Rating Scale, *EQ-Vas* Euroquol-5D visual analog scale, *PSS* perceived stress scale, *LTE-Q* list of threatening experiences questionnaire, *CTQ-SF* childhood trauma questionnaire-short form, *SDU* standard drink unit.

### Metataxonomic analysis of fecal samples

Samples were submitted to metataxonomic profiling (*n* = 113). Globally, 451 representative OTUs were retrieved from 6,436,207 high-quality-filtered sequences. The phyla *Firmicutes*, *Bacteroidetes*, *Proteobacteria*, *Actinobacteria*, and *Verrucomicrobia* accounted for 99.36% of the sequencing data (Supplementary Fig. S1).

Bacterial diversity (Shannon index) was not different among the different groups (Supplementary Fig. S2) of patients (HC, a-MDD, and r-MDD) (*p* value > 0.05) and no statistical differences were found in relation to the 10 most abundant genera (Supplementary Table S3).

**Fig. 1:**
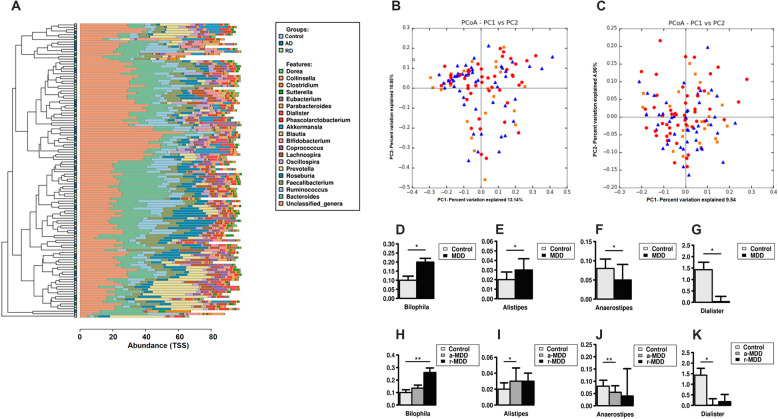
Metataxonomic analysis and abundance of bacteria genera in fecal samples. **A** Comparison of the relative abundance of the 20 most abundant genera (TSS; total-sum normalization) in the three groups of subjects. The dendrogram was based on genera similarity between the samples. Hclust with complete linkage method from the Calypso online software was used to compute the hierarchical clustering. PCoA plots of bacterial profiles based on Bray–Curtis similarity analysis (relative abundance) (**B**) and on the Jaccard’s coefficient for binary data (presence/absence) (**C**) from the three groups of subjects (blue triangle, control group; red circles, a-MDD group; orange squares, r-MDD group). The value given on each axis label represents the percentage of the total variance explained by that axis. Relative abundance of the genera *Bilophila* (**D**), *Alistipes* (**E**), *Anaerostipes* (**F**), and *Dialister* (**G**) in MDD patients compared with HC. Patients with r-MDD presented an increased presence of sequences belonging to the genus *Bilophila* (**H**). Patients with a-MDD showed increased abundance of sequences belonging to the genus *Alistipes* (**I**), while those corresponding to the genera *Anaerostipes* and *Dialister* were decreased (**J**, **K**). Differences in sample group genera were compared by using either the Wilcoxon rank test for variables with two groups or the Kruskal–Wallis test for variables with more than two groups. To correct for multiple comparisons, Bonferroni-adjusted significance levels were set for each analysis. *p < 0.05, **p < 0.01 vs. healthy controls (HC).

There was no clear separation between the subjects according to the relative abundance of the 20 most abundant genera (TSS; total-sum normalization and the Hclust with complete linkage method) (Fig. 1A). At the OTUs level, the PCoA plots of the Bray–Curtis dissimilarity matrix revealed that the samples did not cluster according to their diagnosis (Fig. 1B), while the analysis of similarity (PERMANOVA) showed that there was no statistical difference between the three groups of subjects (*p* = 0.089). The binary Jaccard distance matrix (presence/absence) indicated that samples did not cluster according to the diagnosis (*p* = 0.131) (Fig. 1C).

The abundance of sequences from the genera *Bilophila* and *Alistipes* was higher in MDD patients (both MDD groups combined) compared with HC (Fig. 1D, E). Oppositely, the abundance of sequences from the genera *Anaerostipes* and *Dialister* was lower in MDD patients (Fig. 1F, G).

When the subgroups were analyzed, a-MDD patients showed higher abundance of sequences belonging to the genus *Alistipes* (Fig. 1I) while lower levels of genera *Anaerostipes* and *Dialister* (Fig. 1J, K). Patients with r-MDD presented higher presence of the genus *Bilophila* (Fig. 1H).

### Effects of the MDD on the innate immune pathway

#### MDD patients vs. HC

**Fig. 2:**
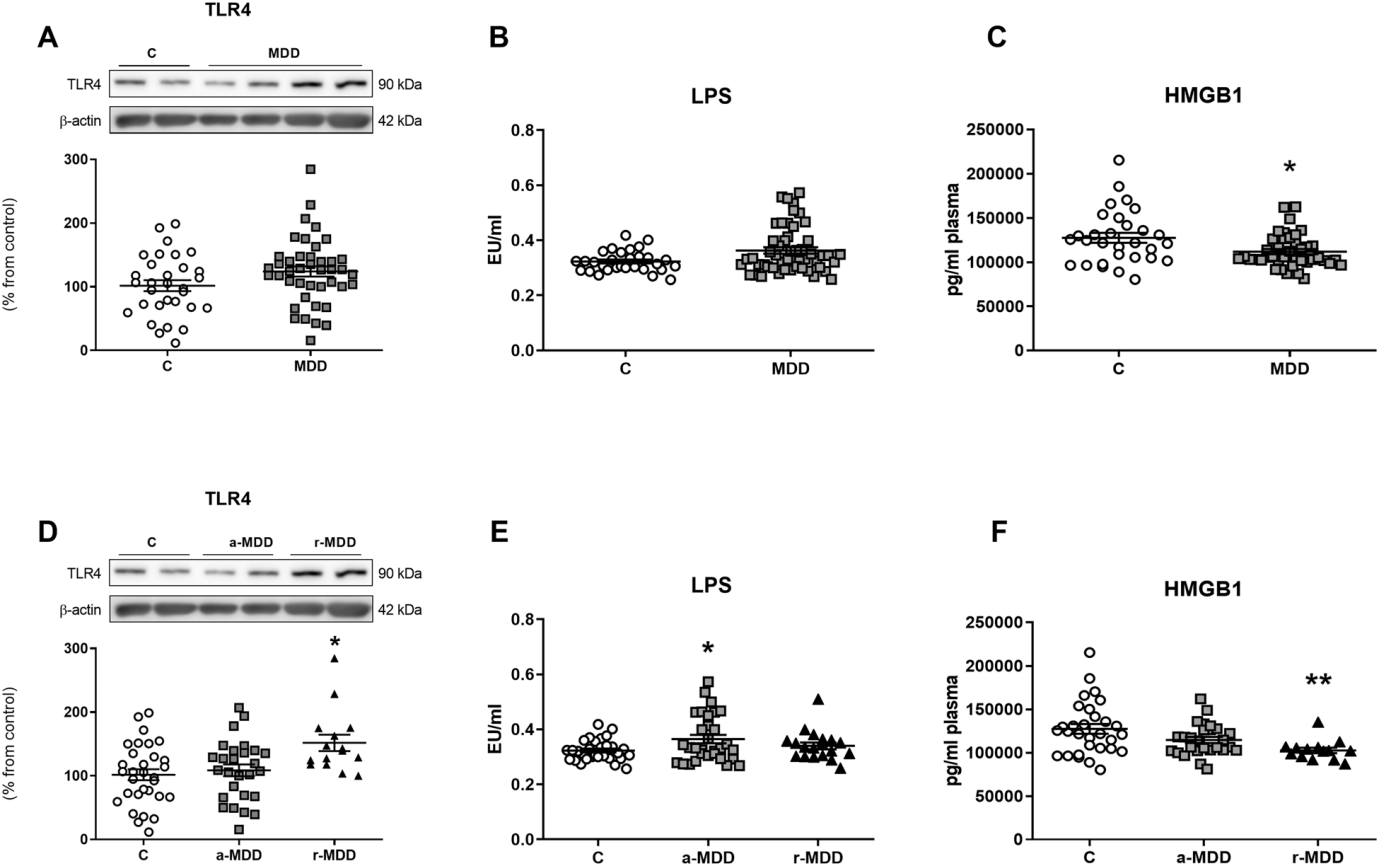
Effects of the MDD on the innate immune pathway. TLR-4 protein expression in PBMCs (**A**) and LPS plasma levels (**B**) were not affected by the MDD. Plasma levels of HMGB1 decreased in the MDD group (**C**). TLR-4 expression was not altered in the a-MDD group and was increased in the r-MDD group compared with HC (**D**). LPS plasma levels augmented in the a-MDD and were not affected in the r-MDD group compared with HC (**E**). HMGB1 plasma levels decreased in the r-MDD group compared with HC (**F**). Data are expressed as mean ± standard error of the mean (SEM). In the western blots the densitometric data of the respective bands of interest are normalized by β-actin (lower band). Samples were from parallel experiments and that gels/blots were processed in parallel. An unpaired two-tailed *t*-test was performed when comparing two groups. For more than two groups comparisons, a one-way ANOVA with a Tukey post-hoc test was employed, and in those cases in which the data did not follow a Gaussian distribution, a nonparametric ANOVA with a Kruskal–Wallis test followed by a Dunn’s post-hoc test was performed. *p < 0.05, **p < 0.01 vs. healthy controls (HC).

The TLR-4 protein expression in PBMCs and LPS plasma levels did not show differences among groups (Fig. 2A, B). However, the plasma levels of the HMGB1 protein were lower in the MDD group (Fig. 2C).

#### Comparison between a-MDD, r-MDD, and HC

The a-MDD group presented not altered expression of TLR-4 (Fig. 2D), higher LPS plasma levels (Fig. 2E), and no changes in the HMGB1 plasma levels (Fig. 2F) compared with HC. The r-MDD group displayed higher TLR-4 expression (Fig. 2D), no changes in LPS plasma levels (Fig. 2E), and lower HMGB1 plasma levels compared with HC (Fig. 2F).

### Effects of MDD on intra- and intercellular inflammatory parameters

#### MDD patients vs. HC

**Fig. 3:**
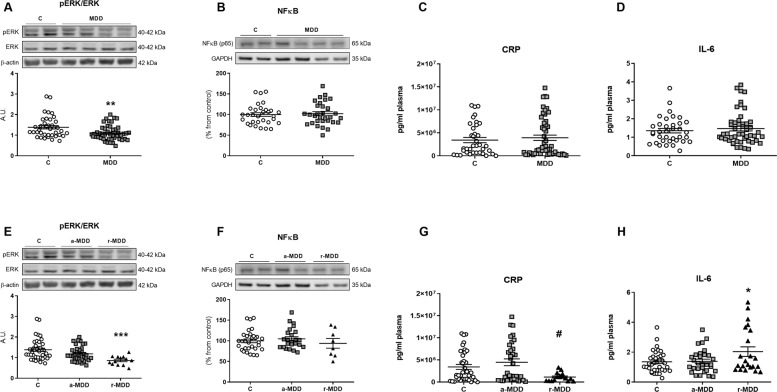
Effects of MDD on intra- and intercellular inflammatory parameters. The ratio between the activated ERK (pERK) and the ERK total form decreased in PBMCs from patients with MDD compared with HC (**A**). Patients with MDD did not show differences in the protein levels of NF-κB nor in the plasma levels of CRP and IL-6 compared with HC (**B**–**D**). a-MDD patients did not show differences when compared with HC (**E**–**H**). pERK/ERK ratio in PBMCs decreased (**E**) and plasma IL-6 increased (**H**) in r-MDD patients compared with HC. Plasma CRP levels in the r-MDD group decreased compared with the a-MDD group (**G**). Data are expressed as mean ± standard error of the mean (SEM). In the western blots the densitometric data of the respective bands of interest are normalized by β-actin or by GAPDH (lower band). Samples were from parallel experiments and that gels/blots were processed in parallel. An unpaired two-tailed *t*-test was performed when comparing two groups. For more than two groups comparisons, a one-way ANOVA with a Tukey post-hoc test was employed, and in those cases in which the data did not follow a Gaussian distribution, a nonparametric ANOVA with a Kruskal–Wallis test followed by a Dunn’s post-hoc test was performed. *p < 0.05, **p < 0.01, ***p < 0.001 vs. healthy controls (HC); ^#^*p* < 0.05 vs. a-MDD.

The ratio between the activated (phosphorylated) ERK and the ERK total form was decreased in PBMCs from MDD patients compared with HC (Fig. 3A).

Patients with MDD did not show differences in the protein levels of the nuclear factor *kappa*B (NFκB) nor in the plasma levels of C-reactive protein (CRP) and interleukin (IL)-6 compared with HC (Fig. 3B–D).

#### Comparison between a-MDD, r-MDD, and HC

Patients with a-MDD did not show changes when compared with HC in any of the intra- and intercellular inflammatory parameters analyzed (Fig. 3E–H). Patients with r-MDD presented lower levels of the pERK/ERK ratio in PBMCs (Fig. 3E) and higher plasma levels of IL-6 (Fig. 3H) compared with HC. The r-MDD group presented lower plasma CRP levels compared with the a-MDD group (Fig. 3G).

### Effects of the MDD on the cyclooxygenase-2 pathway, on the oxidative/nitrosative response, and on counterbalancing mechanisms

#### MDD patients vs. HC

**Fig. 4:**
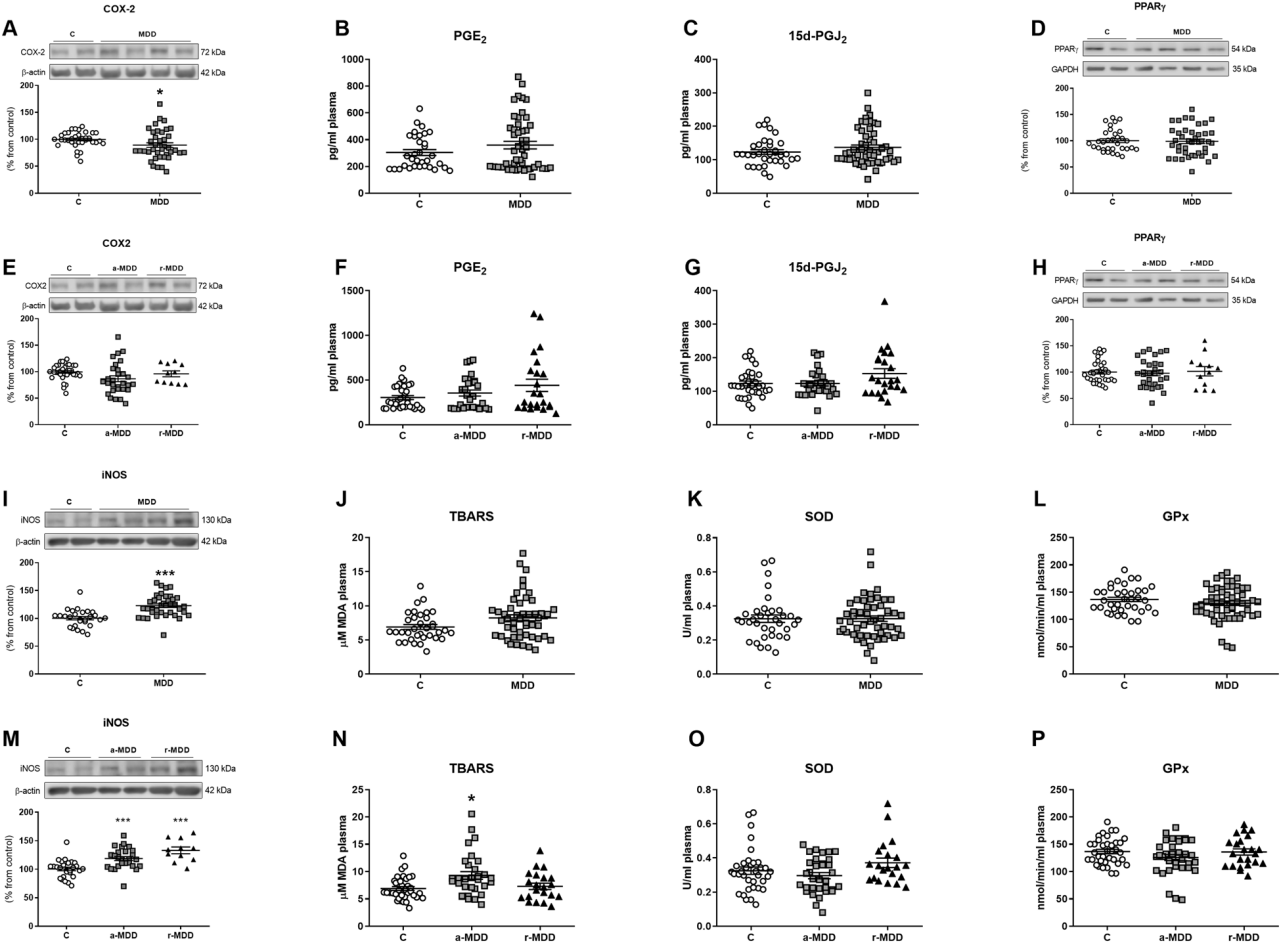
Effects of the MDD on the cyclooxygenase-2 pathway, on the oxidative/nitrosative response, and on counterbalancing mechanisms. Patients with MDD presented lower levels of COX-2 expression in PBMCs (**A**) compared with HC, without changes in PGE_2_ (**B**) and in 15d-PGJ_2_ (**C**) plasma levels. The MDD did not affect the PPARγ expression (**D**). The a-MDD and r-MDD groups did not show any difference between them nor with the HC group (**E**–**H**). The MDD increased iNOS protein levels in PBMCs (**I**) but no affected the activity of TBARS, SOD and GPx (**J–L**). a-MDD and r-MDD groups presented increased iNOS expression in PBMCs (**M**). The a-MDD group shown increased TBARS levels compared with HC (**N**). There were no differences in the activity of SOD and GPx among groups (**O**, **P**). Data are expressed as mean ± standard error of the mean (SEM). In the western blots the densitometric data of the respective bands of interest are normalized by β-actin or by GAPDH (lower band). Samples were from parallel experiments and that gels/blots were processed in parallel. An unpaired two-tailed *t*-test was performed when comparing two groups. For more than two groups comparisons, a one-way ANOVA with a Tukey post-hoc test was employed, and in those cases in which the data did not follow a Gaussian distribution, a nonparametric ANOVA with a Kruskal–Wallis test followed by a Dunn’s post-hoc test was performed. *p < 0.05, ***p < 0.001 vs. healthy controls (HC).

MDD patients presented lower levels of COX-2 expression in PBMCs (Fig. 4A) compared with HC, without changes in the proinflammatory PGE_2_ and the anti-inflammatory 15d-PGJ_2_ plasma levels (Fig. 4B, C). The anti-inflammatory nuclear factor PPARγ expression was not affected between the different groups (Fig. 4D). Moreover, MDD patients displayed higher isoform of the nitric oxide synthase (iNOS) protein levels in PBMCs (Fig. 4I) but no changes when analyzing TBARS, SOD and GPx enzymatic activities (Fig. 4J–L).

#### Comparison between a-MDD, r-MDD, and HC

The COX-2 pathway was not altered in the a-MDD and r-MDD groups not showing any difference between them nor with the HC group (Fig. 4E–H).

Both a-MDD and r-MDD groups presented higher iNOS expression in PBMCs (Fig. 4M). The a-MDD group shown higher TBARS levels compared with HC (Fig. 4N). There were no differences in the activity of SOD and GPx among groups (Fig. 4O, P).

## Discussion

This study presents evidence about differences in some bacterial genera and changes in the innate immune response that could be associated to MDD diagnosis. Patients in an active depressive episode, with higher depressive scores and lower health-related quality of life, showed some different profile in gut microbiota that could be linked to the changes in the inflammatory markers also detected in this group compared to patients with a depressive episode in remission or recovered from a depressive episode. Disentangling these patterns of variations and their relationships with different phases of the illness could be particularly valuable to understand further neurobiological mechanisms involved in MDD.

Fecal samples did not cluster according to the presence or the absence of MDD. In addition, bacterial diversity was not different among the groups. A recent systematic review has revealed the existence of a wide disparity of results among human case–control studies on the relationships between MDD and fecal microbiota [44]. Therefore, there is not a minimal consensus regarding neither microbial diversity, relative abundance, nor directionality of differences in taxa associated with MDD. Such heterogeneity of results may be due to differences in the studied populations as well as the different methodologies used in the different studies. Our study comprised a white Caucasian population, so further research is warranted, and a definitive conclusion about the effects of MDD in bacterial diversity cannot be reached at present.

Genera *Bilophila* (phylum *Proteobacteria*) and *Alistipes* (phylum *Bacteroidetes*) were higher and *Anaerostipes* and *Dialister* (phylum *Firmicutes*) were lower in the feces of patients with MDD when compared with HC. Furthermore, sequences corresponding to *Alistipes* were higher, while those of *Anaerostipes* and *Dialister* were lower in a-MDD patients. Finally, r-MDD patients presented higher abundance of *Bilophila* compared with HC. To our knowledge, this is the first report, showing a higher *Bilophila* abundance in fecal samples from patients recovered from MDD. A higher amount of *Alistipes* had been previously found in relation to MDD patients [29, 45].

*Bilophila* and *Alistipes* are Gram-negative bacteria, and consequently, the LPS from their membrane can stimulate the innate immune system via TLR-4 activation [12] after an intestinal dysfunction allowing bacterial translocation. Importantly, TLR-4 activation induces depressive-like behaviors in animal models, and it has been proposed as a crucial factor in the inflammatory theory of depression [9, 23]. Here, plasma LPS levels are higher in the group with the worse clinical condition (a-MDD), and thus, the higher abundance of *Bilophila* and *Alistipes* could be responsible for the effects on the immune response detected in this group.

There are other mechanisms through which these two bacterial genera may be involved in MDD, one of them being a possible relationship of *Bilophila* with mood-related disorders. In particular, mice subjected to nerve injury presented an anhedonia-like phenotype and increased stool levels of this genus [46]. Another conceivable mechanism would be based in the fact that *Alistipes* species are indole-positive and, therefore, may affect tryptophan availability [47]. As tryptophan is the precursor of serotonin (5-HT), higher levels of *Alistipes* may disrupt the serotonergic system balance.

*Bilophila* presented higher levels in r-MDD and *Alistipes* in a-MDD patients, compared with HC, indicating that some elements of the microbiota are changing through the MDD course with a possible shift toward a percentage increase in the Gram-negative bacterial ratio, although we cannot infer this presumed shift based in our results and further research is needed. In this case, a Gram-negative genus related to the impairment of the serotonergic system (i.e., *Alistipes*) would be higher in the active phase of MDD, while a Gram-negative genus associated with prolonged pathologies (i.e., *Bilophila*), including inflammatory bowel disease (IBD), would be higher in the r-MDD patients.

*Anaerostipes* and *Dialister* showed lower levels in MDD compared with HC. *Anaerostipes* is a Gram-positive anaerobic genus from the phylum *Firmicutes*. *Anaerostipes* species can metabolize carbohydrates producing butyrate, a short-chain fatty acid that plays a key role for gut homeostasis [48, 49] with immunosuppressive and anti-inflammatory functions [44]. Thus, the lower *Anaerostipes* abundance could explain the effects on the inflammatory and oxidative/nitrosative stress responses detected in MDD. Moreover, *Anaerostipes* improves depressive-like behaviors in stressed mice and through an improvement of the serotonergic system balance and increasing trophic factors expression [50]. The lower levels of *Anaerostipes* observed in our patients with MDD could be related to a critical mechanism involved in the pathophysiology of MDD at the microbiota level. However, we are still far from being able to establish causality, and further research is warranted.

*Dialister* is a Gram-negative genus from the phylum *Firmicutes*. The relative significance as well as broad microbiological data on *Dialister* species in human clinical samples remains rather scarce. It has been positively correlated with spondyloarthritis activity [51]. Some *Dialister* species are capable of generating both acetate and propionate, and in Crohn’s disease, a condition in which there is an increased permeability of the intestinal mucosal barriers allowing bacterial translocation, a decrease of *Dialister* has been reported [52]. Curiously, a recent study has shown that *Dialister* is depleted in MDD, even after correcting for the confounding effects of antidepressants, and that is positively associated with quality-of-life indicators [53]. In fact, *Dialister* was considered as a potential *psychobiotic* genus and, consequently, as a main target for follow-up research. Our data show a *Dialister* depletion in MDD, which would agree with the view of a genus negatively associated with depression and positively associated with a healthy condition for the host.

*Anaerostipes* and *Dialister* declined in a-MDD patients. A previous report found that *Dialister* is relatively more abundant in HC than in a-MDD patients [29]. This reduction in a-MDD could be supporting the protective nature of these genera against depression, through mechanisms mentioned above (e.g., immunosuppression and prevention of bacterial translocation) and clearly deserves further consideration. *Dialister* was not completely depleted in r-MDD suggesting a protective nature of this genus.

We aimed to recognize the potential role of TLR-4 in our scenario as one of the predominant sources of the TLR-4 activation is the altered gut microbiota [21] and the subsequent shifts in microbiota through increased intestinal barrier permeability that has been suggested in patients with MDD [27, 54].

MDD patients present lower plasma levels of HMGB1, an important mediator in innate immunity, characterized as an alarmin or danger signal involved in the LPS-induced depressive-like behavior [55]. The reduction in the HMGB1 levels could seem counterintuitive and contrary to the inflammatory hypothesis; however, when splitting the groups, this reduction is mostly accounted by the low levels detected in the r-MDD group. Hence, reduced HMGB1 levels among our MDD patients could be related to the recovery process. Further research is necessary, but the HMGB1 decrease could be explaining the higher TLR-4 expression detected in r-MDD; it is plausible that as the plasma levels of the ligand (HMGB1) are dropping, the expression levels of the receptor are climbing, as a compensatory mechanism. However, the LPS raise detected in patients with a-MDD could also cause the TLR-4 expression increment: r-MDD patients have been a-MDD formerly, and thus, the higher LPS levels during their a-MDD period could be activating the TLR-4, causing the TLR-4 increment detected in their r-MDD phase.

LPS presented higher levels only in a-MDD compared with HC. LPS from Gram-negative bacteria is the main ligand of the TLR-4 and a parameter widely related to depressive-like behavior [9]. A possible cause for this increment could be the dysfunction of the intestinal barrier and the ensuing bacterial translocation. Interestingly, there are higher levels of some Gram-negative genera (i.e., *Alistipes*) in the stool of the a-MDD patients.

In MDD patients the ratio pERK/ERK in PBMCs presented lower values compared with HC. The r-MDD group presented lower levels of the pERK/ERK ratio in PBMCs and plasma CRP and higher plasma levels of IL-6. ERK is a downstream element activated after TLR-4 stimulation, implicated in numerous signaling cascades wherein various extracellular stimuli induce inflammation [13]. Several studies associate the ERK cascade with the etiology and treatment of depression [56]. Postmortem brain studies indicate that individuals who committed suicide have ERK abundance and activity. Conversely, in experimental studies the ERK cascade is activated by antidepressants, and apparently, its antidepressant effects involve neurotrophic and growth factors [57]. Here, the lower ERK levels in MDD patients could be the result of its expression levels in the r-MDD group, which is the group with a better clinical condition. It could be possible that antidepressant treatments have stimulated this pathway and when achieving the response phase, this pathway had been exhausted. In addition, it could be possible that the lessening of this MAPK is resulting from the achievement of a better clinical condition. Whatever the case may be, further research is needed to fully understand the role of ERK in the MDD.

CRP has been measured in numerous prior studies of MDD [58]. There is heterogeneity of effect size between studies that may be attributable to clinical variability, with higher CRP in severe depression than in mild/moderate depression [59]. High levels of CRP are associated with antidepressant treatment resistance and lower remission rates [60]. Our data showed lower CRP levels in r-MDD. This would agree with this view of CRP as an indicator of current severity and a potential predictor of antidepressant response.

Meta-analyses indicate that IL-6 levels are elevated in the blood of MDD patients [2, 58], suggesting that IL-6 levels might serve as a predictive biomarker. As IL-6 acts on so many diverse tissues throughout the organism, dysregulation of this cytokine can precipitate a multitude of events relevant to depression [61]. Importantly, IL-6 has many functions within the immune system depending on the type of organ it is acting upon and its signaling is complex and resulting in both inflammatory and anti-inflammatory cascades [62]. Our results show higher IL-6 levels in the r-MDD group compared with HC. A possible explanation could be the actions of the antidepressants, as in vitro studies employing whole blood cultures from MDD patients show that some antidepressants induce an increment in the peripheral levels of several cytokines, including IL-6 [63]. Another possibility could be that the IL-6 detected would be acting as anti-inflammatory through its classical signaling.

The inducible iNOS expression is higher in MDD patients compared with HC. This increment is maintained in both a-MDD and r-MDD groups, but the TBARS, a marker of damage produced by oxidative stress, is only higher in the a-MDD group, and the two antioxidant enzymes analyzed were not affected by the disease.

Oxidative/nitrosative stress is increased in MDD [64] and it can be a final consequence of the inflammation detected in this disease [65]. Preclinical research shows that the oxidative/nitrosative stress caused by translocated bacteria, as well as the parameters involved in this stress regulation, are implicated in the actions of a depression model on the CNS and the depressive-like behavior [25]. More research is warranted, but oxidative/nitrosative stress appears to be a crucial mechanism through which the inflammation caused by the potential bacterial translocation would be affecting the clinical evolution of MDD patients. Similarly, more factors involving the antioxidant machinery should be studied.

This study has some limitations. One is the number of samples caused by the difficulties to obtain a high number of this kind of samples from well-characterized patients. This limitation could explain the absence of significant results when the bacteria diversity was analyzed. Anyhow, the number of samples allowed to get enough statistical power to discover differences in bacteria genera and in biochemical parameters. Another limitation is the absence of a clear way to relate the theoretical bacterial translocation with the immune system, although the plasma LPS levels and the TLR-4 activation in PBMCs could be considered indirect indicators. Microbiota composition is influenced by other factors such as lifestyle and medication use, and this is also a limitation. Finally, there is a common limitation to the microbiota and CNS studies, namely that the practical elucidation of metagenomes in a MGB axis context persists challenging and is hampered by the lack of a dedicated reference database of gut microbial neuroactive metabolic potential [53].

In summary, there are bacterial genera with potential implications in the pathophysiology of depression, whose abundance is altered in the feces of MDD patients. Besides, a-MDD and r-MDD patients present differences among them that are worthy of further consideration, being the a-MDD patients the group showing the most noticeable changes. In addition, bacterial translocation (inferred by the increment in the plasma levels of LPS) could be affecting the immune response, and the oxidative/nitrosative stress seems to be a crucial mechanism through which the inflammation would be affecting the clinical evolution of MDD patients. It is not clear whether stimulation of the immune parameters associated with MDD is a precipitating event of the disorder or a process within MDD. However, data seem to point to an inflammatory process in those patients with a more severe condition, recurrent symptoms, and/or treatment resistance. Thus, identifying compositional microbiota markers characteristic for MDD patients and their links with the potential inflammatory states present in those patients could represent a promising therapeutic approach, especially considering that dietary manipulations can have an impact on the gut microbiota, potentially facilitating therapeutic interventions in some MDD patients [66].

## Supplementary information

Supplemental Materials and Methods, Tables and Figures
