# Supplementary material for: Gut microbiota, innate immune pathways, and inflammatory control mechanisms in patients with major depressive disorder
Source: Transl Psychiatry. 2021 Dec 21;11:645. doi: 10.1038/s41398-021-01755-3 (PMC8692500; doi:10.1038/s41398-021-01755-3)
Supplement: Supplementary file 1 — Supplemental Materials and Methods, Tables and Figures [file 41398_2021_1755_MOESM1_ESM.doc]

**Supplemental Materials and Methods, Tables and Figures.**

*Subject inclusion and exclusion criteria:*

These criteria were pre-established. Briefly, for the patients with MDD group the inclusion criteria were: (1) DSM-IV-TR diagnosis of MDD; (2) age between 18 and 65 years; and (3) written informed consent given. The most important exclusion criteria were: (1) past or current comorbidity with other severe mental disorders (e.g., bipolar disorder, schizophrenia, schizoaffective disorder); (2) somatic comorbidity which could interfere with the status of the study's immuno-inflammatory biomarkers (acute infection, fever, acute allergies, cancer, autoimmune diseases or chronic conditions); (3) use of drugs of abuse or treatment with antibiotics, immunosuppressive drugs or vaccines within the past 6 months prior to enrollment inclusion in the study; (4) treatment with anti-inflammatory drugs within the seven days prior to blood collection; and (5) ingestion of probiotics two days before stool samples.

Ethical approval was obtained from Hospitals’ Ethics Committees. All participants signed written informed consents after receiving a complete description of the study. The authors assert that all procedures contributing to this work comply with the ethical standards of the relevant national and institutional committees on human experimentation and with the Helsinki Declaration of 1975, as revised in 2008.

*Subjects:*

Sixty-eight patients with MDD were included in the study. This sample was divided into two groups for additional analyses: (1) patients with a current active depressive episode according to DSM-IV-TR and a Hamilton Depression Rating Scale (HDRS) ≥14 (a-MDD; n=46), and (2) patients with MDD who had responded to treatment and remained in remission or with only mild symptoms (HDRS 8-14) (r-MDD; n=22). The mean age was 43.98 years and 77.77% females and 46 patients were a-MDD (mean age 42.1, 78.26% females) and 22 r-MDD (mean age 45.85, 77.27% females). These groups were compared with 34 healthy controls (HC) (mean age 44.72 years and 75.5% females). There were no statistically significant differences among the groups in age nor in gender. See Table 1 for more characteristics of the sample.

*Sample collection and DNA extraction:*

Fecal samples were collected in a sterile plastic cup after the participants completed the clinical assessments and were kept in an icebox. Samples for bacterial genomic DNA extraction were delivered to the laboratory and stored at 80 C. Microbial DNA was extracted from fecal aliquots (200 mg) using the QIAamp© DNA Stool Mini Kit (Qiagen, Hilden, Germany) according to the manufacturer’s instructions, with the additional glass-bead beating steps on a Mini-beadbeater (FastPrep; Thermo Electron Corp., Boston, MA, USA). DNA was quantified using a NanoDrop ND-1000 spectrophotometer (Thermo Electron); integrity and size were assessed by 1.0% agarose gel electrophoresis on gels containing 0.5 mg/mL ethidium bromide. DNA was stored at 20º C before analysis.

*PCR amplification and sequencing:*

16S rDNA gene amplicons were amplified following the Illumina protocol for 16S rDNA gene Metagenomic Sequencing Library Preparation (Part # 15044223 Rev. A). The forward primer (TCGTCGGCAGCGTCAGATGTGTATAAGAGACAGCCTACGGGNGGCWGCAG) and the reverse one (TACGGTAGCAGAGACTTGGTCTGACTACHVGGGTATCTAATCC) were used as previously described (33), generating amplicons targeting the V3-V4 hypervariable region of the 16S rDNA gene. An aliquot of the microbial DNA from each sample (5 ng/μL in 10 mM Tris pH 8.5) was used to initiate the protocol. Libraries were sequenced using a 2x300pb paired-end run (MiSeq Reagent kit v3, MS-102-3001) on a MiSeq Sequencer according to manufacturer’s instructions (Illumina, USA).

*Bioinformatic analysis:*

The bioinformatic analysis was conducted combining R (v 3.2.3), QIIME pipelines (v 1.8.0) (37) and Calypso (v 8.84) (38). Estimates of intrasample diversity were made at a rarefaction depth of 27,000 reads per sample. Alpha diversity was assessed with the Shannon diversity index (SDI), which considers the number and evenness of microbial species, using Kruskal-Wallis. Beta diversity was studied using the principal coordinates analysis (PCoA) to visually display patterns of beta diversity through a distance matrix containing a dissimilarity value for each pairwise sample comparison. For the quantitative and qualitative analyses, the Bray-Curtis and binary Jaccard indices were used, respectively. The PERMANOVA analysis with 999 permutations reveals statistically significant differences (p<0.05). Differences in sample group genera were compared by using either the Wilcoxon rank test for variables with 2 groups or the Kruskal-Wallis test for variables with more than 2 groups. To correct for multiple comparisons, Bonferroni-adjusted significance levels were set for each analysis.

*Specimen collection and preparation:*

Venous blood samples (10 mL) were collected between 8:00 and 10:00 h after overnight fasting. Blood tubes were centrifuged (641xg for 10 min at 4ºC). The resulting plasma samples were collected and stored at -80ºC. The rest of the sample was 1:2 diluted in culture medium (RPMI 1640, LifeTech) and a gradient with Ficoll-Paque (GE Healthcare) was used to isolate mononuclear cells by centrifugation (800 xg for 40 min at room temperature [RT]). The peripheral blood mononuclear cells (PBMC) layer was aspired, suspended in RPMI and centrifuged (1116 xg for 10 min at RT). The supernatant was removed, and the mononuclear cell-enriched pellet was stored at -80ºC.

*Determinations in plasma:*

LPS levels were determined by using a LAL Chromogenic Endpoint Assay (Hycult Biotech, The Netherlands). Interleukin 6 (IL-6), C-Reactive Protein (CRP) and High Mobility Group Box 1 (HMGB1) levels were measured by using the ELISA kits human IL-6 (Diaclone, France), human CRP (RayBiotech, USA) and HMGB1 (Elabscience Biotechnology Co., China), respectively. Activity levels of the antioxidant enzymes superoxide dismutase (SOD) and glutathione peroxidase (GPx) were quantified employing colorimetric activity kits (Cayman Europe, Estonia). Levels of COX by-products prostaglandin (PG) E_2_ and 15-deoxy-∆^12,14^- PGJ_2_ (15d-PGJ_2_) were measured by enzyme immunoassays (PGE_2_ and 15d-PGJ_2_ ELISA kits, Enzo Life Sciences, Switzerland). Finally, lipid peroxidation was determined by the Thiobarbituric Acid Reactive Substances (TBARS) assay (Cayman Europe, Estonia).

*Determinations in PBMCs (Western blot):*

After determining and adjusting protein levels, cytosolic and nuclear extracts were mixed with *Laemmli* sample buffer (Bio-Rad, Hercules, CA) with β-mercaptoethanol. Samples were loaded and the proteins size- separated in 10% SDS-polyacrylamide gel electrophoresis (90V). Proteins from the gels were blotted onto a nitrocellulose membrane using a semi-dry transfer system (Bio-Rad) and were incubated with specific antibodies: (1) iNOS, rabbit polyclonal antibody dilution of 1:750 in 1% BSA (sc651, Santa Cruz Biotechnology); (2) COX-2, goat polyclonal antibody dilution of 1:750 in 2.5% BSA (sc-1747, Santa Cruz Biotechnology); (3) phospho- extracellular-signal-regulated kinase (ERK), rabbit polyclonal antibody dilution of 1:1000 in TBS-tween (8544, Cell Signaling); (4) ERK, rabbit polyclonal antibody dilution of 1:2000 in TBS-tween (4695, Cell Signaling); (5) NFκB p65 rabbit polyclonal, dilution 1:1000 in TBS tween (sc372, Santa Cruz Biotechnology); (6) PPARγ rabbit polyclonal, dilution 1:1000 in TBS tween (sc7196, Santa Cruz Biotechnology); (7) TLR4, goat polyclonal antibody dilution of 1:1000 in 0.5% BSA (sc-16240, Santa Cruz Biotechnology); (8) β-actin mouse monoclonal in a dilution 1:10000 in TBS-tween (A5441, Sigma, Spain); (9) GAPDH mouse monoclonal antibody in a dilution of 1:5000 in TBS-tween (G8795; Sigma, Spain). After washing with a TBS-Tween solution, the membranes were incubated with the respective horseradish peroxidase-conjugated secondary antibodies for 90 minutes at RT and revealed by ECL™-kit following manufacturer's instructions (Amersham Ibérica, Spain). Blots were visualized using an Odyssey® Fc System (Li-COR Biosciences) and quantified by densitometry (NIH ImageJ® software). All blots were performed at least three times in separate assays and densitometries were expressed in percentage from the control group. Several exposition times were analyzed to ensure the linearity of the band intensities. The loading controls were β-actin for the cytosolic fraction and GAPDH for the nuclear fraction (blots shown in the respective figures). Protein levels were measured using Bradford method based on the principle of protein-dye binding.

**Table S1.** Antibodies employed and their unique identifiers provided by the Resource Identification Portal

| **Antibodies (references)** | **Unique Identifiers** |
| --- | --- |
| iNOS (Santa Cruz, sc-651) | RRID:AB_2298577 |
| COX2 (Santa Cruz, sc-1747) | RRID:AB_2084976 |
| pERK (Cell Signaling, #8544) | RRID:AB_11127856 |
| ERK (Cell Signaling, #4695) | RRID:AB_390779 |
| NF-κB/p65 (Santa Cruz, sc-372) | RRID:AB_632037 |
| PPARy (Santa Cruz, sc-7196) | RRID:AB_654710 |
| TLR4 (Santa Cruz, sc-16240) | RRID:AB_2205143 |
| b-actin (Sigma-Aldrich, #A5441) | RRID:AB_476744 |
| GAPDH (Sigma-Aldrich, #G8795) | RRID:AB_1078991 |

**Table** **S2.** Statistical details of the different ANOVAs performed

|  | **3 group** |
| --- | --- |
| **TLR4** | F(2,73)=8.501, p=0.01; Fig 2D |
| **LPS** | F(2,77)=3.334, p=0.04; Fig 2E |
| **HMGB1** | F(2,68)=5.246, p=0.005; Fig 2F |
| **Ratio pERK/ERK** | F(2,85)=15.77, p=0.0004; Fig 3E |
| **NFκB** | F(2,60)=0.59, p=0.55; Fig 3F |
| **CRP** | F(2,85)=4.61, p=0.01; Fig 3G |
| **IL6** | F(2,85)=3.79, p=0.02; Fig 3H |
| **COX-2** | F(2,73)=2.775, p=0.06; Fig 4E |
| **PGE2** | F(2,85)=1.026, p=0.59; Fig 4F |
| **15dPGJ2** | F(2,85)=2.721, p=0.25; Fig 4G |
| **PPARγ** | F(2,71)=0.1162, p=0.89; Fig 4H |
| **iNOS** | F(2,73)=15.60, p<0.0001; Fig 4M |
| **TBARS** | F(2,85)=7.477, p=0.02; Fig 4N |
| **SOD** | F(2,85)=2.57, p=0.08; Fig 4O |
| **GPx** | F(2,85)=1.63, p=0.2; Fig 4P |

**Table S3.** **Bacterial composition at the Phyla and genera levels in the three groups of subjects.**

| ***Phyla//Genera*** | **Control** | | **a-MDD** | | **r-MDD** | | **p-value** ^b^ |
| --- | --- | --- | --- | --- | --- | --- | --- |
|  | **N (%)** ^a^ | **Median % (Q1-Q3)** | **N (%)** | **Median % (Q1-Q3)** | **N (%)** | **Median % (Q1-Q3)** | **(Kruskal-wallis)** |
| ***Firmicutes*** | 49 (100%) | 63.36% (57.7-68.67) | 48 (100%) | 63.32% (59.73-69.86) | 23 (100%) | 64.13% (54.82-69.4) | 0.83 |
| *Ruminococcus* | 49 (100%) | 5.75% (3.23-8.05) | 48 (100%) | 6.27% (3.62-10.00) | 23 (100%) | 7.19% (3.88-10.51) | 0.77 |
| *Faecalibacterium* | 49 (100%) | 6.43% (4.76-8.65) | 48 (100%) | 5.89% (3.12-8.07) | 23 (100%) | 6.99% (3.22-10.34) | 0.37 |
| *Roseburia* | 49 (100%) | 5.64% (2.81-10.99) | 47 (97.92%) | 5.01% (1.50-10.57) | 23 (100%) | 5.11% (2.13-7.78) | 0.66 |
| *Oscillospira* | 49 (100%) | 2.29% (1.58-3.37) | 48 (100%) | 2.97% (1.80-4.01) | 23 (100%) | 2.44% (1.42-4.00) | 0.49 |
| *Lachnospira* | 48 (97.96%) | 2.58% (0.82-4.82) | 48 (100%) | 1.74% (0.94-3.81) | 23 (100%) | 1.91% (0.57-3.78) | 0.45 |
| *Coprococcus* | 49 (100%) | 1.87% (0.88-4.02) | 48 (100%) | 1.53% (0.95-3.27) | 23 (100%) | 1.15% (0.52-3.34) | 0.29 |
| ***Bacteroidetes*** | 49 (100%) | 27.42% (21.42-32.89) | 48 (100%) | 26.77% (21.63-31.51) | 23 (100%) | 26.07% (20.11-34.94) | 0.9 |
| *Prevotella* | 47 (95.92%) | 1.65% (0.01-9.42) | 45 (93.75%) | 3.11% (0.02-7.91) | 21 (91.3%) | 1.30% (0.01-9.72) | 0.84 |
| *Bacteroides* | 49 (100%) | 16.37% (9.25-20.56) | 48 (100%) | 15.23% (10.14-18.20) | 23 (100%) | 14.45% (6.63-22.54) | 0.72 |
| ***Actinobacteria*** | 49 (100%) | 2.08% (1.19-4.45) | 48 (100%) | 1.72% (0.99-4.38) | 23 (100%) | 2.03% (0.78-3.91) | 0.91 |
| *Bifidobacterium* | 47 (95.92%) | 1.28% (0.41-3.08) | 48 (100%) | 1.09% (0.60-2.84) | 23 (100%) | 1.11% (0.23-2.07) | 0.88 |
| ***Proteobacteria*** | 49 (100%) | 1.65% (1.25-2.69) | 48 (100%) | 1.71% (1.36-3.22) | 23 (100%) | 3% (1.68-4.69) | 0.06 |
| ***Verrucomicrobia*** | 40 (81.63%) | 0.18% (0.01-1.33) | 39 (81.25%) | 0.05% (<0.01-2.24) | 19 (82.61%) | 0.61% (0.04-1.69) | 0.33 |
| *Unclassified_genera* | 49 (100%) | 26.38% (21.88-32.11) | 48 (100%) | 25.71% (20.80-33.48) | 23 (100%) | 22.05% (15.91-30.09) | 0.11 |

^a^ N: Number of samples where the phyla or genera were detected and its frequency of detection.

^b^ Kruskal-Wallis Rank Sum Tests - p-value adjusted P (Bonferroni).

**
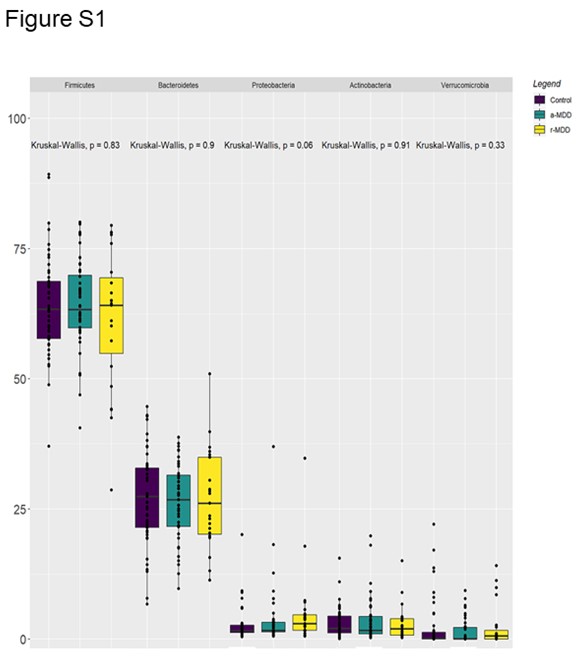
**

**Figure S1.** Bacterial composition at the Phyla level of the 3 groups of subjects. Graphs show the relative abundance of the 5 most abundance Phyla.


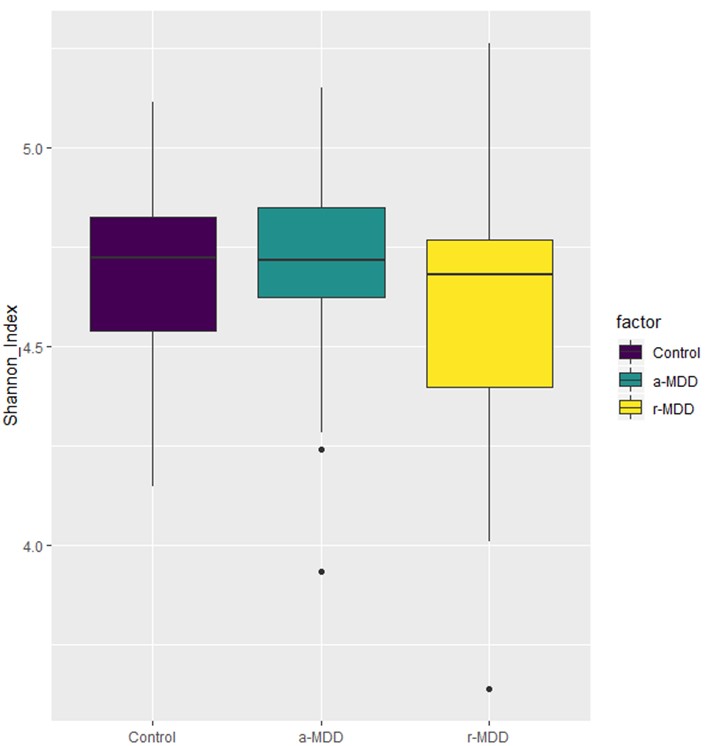
**Figure S2**

**Figure S2.** Boxplot of the changes of Shannon diversity index equitability (evenness) for each group of subjects.
